# Parliament and Publications

**Published:** 1911-04-15

**Authors:** 


### Parliament and Publications.

### NOTIFICATION OF MEASLES.

Mr. John Burns has announced that he is considering the advisability of making measles a notifiable disease.

### SMALL-POX IN LONDON.

During the four weeks ending April 4, said Mr. Burns in reply to a question in the House of Commons, only ten small-pox patients were removed to the hospitals of the Metropolitan Asylums Board.

### REPORT OF THE CHARITY COMMISSIONERS.

The fifty-eighth report of the Charity Commissioners states that of the million and a half given without trust for investment last year the amount for medical charities was £602,840. The amount given under trusts for investment for medical charities which came under the notice of the Commissioners was £254,701. "Charity Commissioners," Cd. 5573. Price 2½d.

### MEDICAL PRACTITIONERS AND THE PETROL DUTY.

Mr. Ginnell has again questioned the Chancellor of the Exchequer regarding the rebate of the motor spirit duty, and Mr. Hobhouse, in reply, stated that wherever satisfactory evidence is forthcoming that the duty has been paid by a duly qualified medical practitioner for the purpose of his profession, repayment of the proper amount is made.

### SANITARY AUTHORITIES AND THE SUPPLY OF VACCINE LYMPH.

In reply to a question by Mr. Snowden, Mr. John Burns stated that there is no provision in the general law enabling sanitary authorities to supply vaccine lymph or to pay for vaccinations, but in some few cases sanitary authorities have been empowered to provide facilities for vaccination by virtue of orders issued by the Local Government Board under the Public Health Act.

### CANCER RESEARCH.

The report of Dr. E. F. Bashford, General Superintendent of Research and Director of the Laboratory of the Imperial Cancer Research Fund, on the Second International Conference on Cancer Research held at Paris in October last, has been published as a Parliamentary Paper. "Cancer Research," Cd. 5590. Price ½d.

### HEALTH VISITORS.

Replying to Mr. Charles Bathurst, Mr. Burns said he was unable to state in how many cases women had been appointed to perform the functions of health visitors by County and District Councils, but in several instances officers, such as inspectors of midwives, school nurses, and assistant inspectors of nuisances had been utilised to give advice as to the nurture and care of infants.

### PLAGUE IN INDIA.

Replying to Mr. Keir Hardie, the Under-Secretary of State for India said that the increased prevalence of plague in certain parts of India at the present time of the year is believed to be due to the fact that this is the season of greatest prevalence of rats and rat fleas. It is a well-recognised measure of precaution that on the first sign of the presence of plague the residents should evacuate their dwellings. The Government and the local authorities assist them to do this by the supply of tents and hutting materials, by erecting temporary dwellings, by providing guards for the vacated houses, and by money rewards.

### DIPHTHERIA.

Questioned concerning an outbreak of diphtheria amongst wood-pigeons at Elton, Hunts, Mr. John Burns explained that so far as research has at present gone he understands that it does not suggest the communicability of pigeon diphtheria to man.

### THE MANUFACTURE OF MEAT EXTRACTS.

Mr. Crumley asked the President of the Local Government Board whether factories in which bovril and other meat extracts are manufactured are regularly inspected to see that sound meat, and meat only, is used in their manufacture. Mr. Burns replied that so far as he is aware these meat extracts are not prepared from meat in this country. Inspectors from his department do not inspect the factories, and the duty of inspecting articles of food intended for sale for human consumption rested on the local authority.

### SHELLFISH AND DISEASE.

A report on the conditions under which certain shellfish other than oysters are grown, collected, cleansed, and stored, and the relation of such treatment to the prevalence of enteric fever and other illness, has been published as a supplement to the report of the Medical Officer of Health to the Local Government Board. The inquiry was conducted by Dr. H. Timbrell Bulstrode, who shows that shellfish are in many instances collected from natural beds which are liable to become specifically polluted by sewage, and that even when these shellfish are procured from localities remote from the risks of pollution they are at times laid down in dangerous proximity to sewer or drain outfalls. Dr. Bulstrode advises that, so long as the state of affairs revealed in his report obtains, those persons who desire to avoid contraction of shellfish-borne enteric fever or gastro-enteritis should either abstain entirely from such shellfish as mussels or cockles or consume them only after they have been actually at the boiling-point for at least five minutes. "Shellfish other than oysters in relation to disease," Cd. 5313. Price 8s.

